# *Staphylococcus aureus* enterotoxins induce FOXP3 in neoplastic T cells in Sézary syndrome

**DOI:** 10.1038/s41408-020-0324-3

**Published:** 2020-05-14

**Authors:** Andreas Willerslev-Olsen, Terkild B. Buus, Claudia Nastasi, Edda Blümel, Maria Gluud, Charlotte M. Bonefeld, Carsten Geisler, Lise M. Lindahl, Maarten Vermeer, Mariusz A. Wasik, Lars Iversen, Jürgen C. Becker, Mads Hald Andersen, Lise M. R. Gjerdrum, Ivan V. Litvinov, Thomas Litman, Thorbjørn Krejsgaard, Anders Woetmann, Niels Ødum

**Affiliations:** 1 Department of Immunology and Microbiology; LEO Foundation Skin Immunology Research Center, University of Copenhagen, Copenhagen, Denmark; 2 Department of Dermatology, Aarhus University Hospital, Aarhus, Denmark; 3 Department of Dermatology, Leiden University Medical Center, Leiden, The Netherlands; 4 Department of Pathology and Laboratory Medicine, University of Pennsylvania, Philadelphia, PA USA; 5 Department of Translational Skin Cancer Research, German Cancer Consortium (DKTK), University Hospital of Essen, Essen, Germany; 6 Deutsches Krebsforschungsinstitut (DKFZ), Heidelberg, Germany; 7 Center for Cancer Immune Therapy, Department of Hematology, Herlev Hospital, University of Copenhagen, Herlev, Denmark; 8 Department of Pathology, Zealand University Hospital, Roskilde, Denmark; 9 Division of Dermatology, McGill University Health Centre, Montreal, QC Canada

**Keywords:** Cancer microenvironment, Tumour immunology

## Abstract

Sézary syndrome (SS) is a heterogeneous leukemic subtype of cutaneous T-cell lymphoma (CTCL) with generalized erythroderma, lymphadenopathy, and a poor prognosis. Advanced disease is invariably associated with severe immune dysregulation and the majority of patients die from infectious complications caused by microorganisms such as, *Staphylococcus aureus*, rather than from the lymphoma per se. Here, we examined if staphylococcal enterotoxins (SE) may shape the phenotype of malignant SS cells, including expression of the regulatory T-cell-associated marker FOXP3. Our studies with primary and cultured malignant cells show that SE induce expression of FOXP3 in malignant cells when exposed to nonmalignant cells. Mutations in the MHC class II binding domain of SE-A (SEA) largely block the effect indicating that the response relies at least in part on the MHC class II-mediated antigen presentation. Transwell experiments show that the effect is induced by soluble factors, partly blocked by anti-IL-2 antibody, and depends on STAT5 activation in malignant cells. Collectively, these findings show that SE stimulate nonmalignant cells to induce FOXP3 expression in malignant cells. Thus, differences in exposure to environmental factors, such as bacterial toxins may explain the heterogeneous FOXP3 expression in malignant cells in SS.

## Introduction

Cutaneous T-cell lymphoma (CTCL) is a heterogeneous group of non-Hodgkin T-cell lymphomas with primary involvement of the skin. Sézary syndrome (SS) is an especially aggressive subtype of CTCL characterized by the presence of leukemic cells in the blood, generalized erythroderma, lymphadenopathy, and a median survival of only 3 years^1–3^. The etiology is unknown and recent attempts to identify recurrent key driver mutations have had limited success, while chromosomal instability and major genetic alterations are commonly seen^4–10^. Importantly, SS is a heterogeneous disease and we recently reported that malignant cells also display a pronounced heterogeneity at the single-cell level in individual SS patients^11^.

A defining feature of SS is that advanced disease is associated with a distinctive tumor microenvironment dominated by T_H_2 cytokines^2,12^ and a general absence of T_H_1 cytokines, such as IFN-γ and IL-12 (refs. ^13–16^). Expression of the immunosuppressive cytokines TGF-*β* and IL-10 has also been reported, and is likely caused by deregulated JAK/STAT3 and NFκB signaling^17–20^. SS patients display severe functional defects in neutrophils, NK−, and dendritic cells, effectively impairing cellular immunity^16,21,22^. Notably, Berger et al. reported that immature dendritic cells in vitro induced a regulatory phenotype in malignant cells with expression of the IL-2 receptor subunit alpha (CD25) and FOXP3 (ref. ^23^). This observation made the authors hypothesize that CTCL involved malignant proliferation of regulatory T (Treg) cells^23^. Expression of FOXP3 in malignant cells is conspicuous as it is a major transcription factor essential in driving the differentiation of Tregs. However, it has been a matter of controversy whether or not malignant cells express FOXP3 and display a Treg phenotype in vivo, and very different frequencies of FOXP3-positive malignant cells have been reported in different cohorts of SS patients^24–29^. Moreover, malignant cells may even display a heterogeneous FOXP3 expression pattern at the single-cell level in an individual patient^30^ or in skin lesions, as judged from immunohistochemistry staining of cells with neoplastic morphology^17^.

As advanced SS is associated with an increasingly impaired immune defense, SS patients have an increased risk of contracting infections^31^ and the majority of patients with advanced disease die from infection rather than from the lymphoma per se^32,33^. Notably, severe bacterial infections are almost exclusively seen long after the diagnosis has been established^34^. Since malignant cells induce structural changes in the skin leading to impairment of the skin barrier in 3D in vitro skin^35^, it is likely that lymphoma-induced skin barrier defects play an important role in the increased susceptibility to bacterial infections in SS.

*Staphylococcus aureus* is a very prevalent pathogen in SS, and accounts for much morbidity and mortality due to recurrent or chronic skin infections, sepsis, pneumonia, and intra-abdominal infections^32,33,36,37^. Some studies have also implicated staphylococcal enterotoxins (SE) from *S. aureus* in the pathogenesis of CTCL. SE can induce activation of STAT3 in malignant cells and secretion of cytokines, such as IL-10 (refs. ^20,38^). Other previous studies have shown that clearing *S. aureus* infections with antibiotics is associated with clinical improvement and a decrease in the tumor burden in CTCL patients (reviewed in ref. ^39^). We recently demonstrated that eradication of *S. aureus* in patients with advanced CTCL by systemic treatment with antibiotics induced a decrease in the malignant T-cell clone, diminished skin inflammation, and led to the clinical improvement in patients with advanced CTCL, providing the first evidence that *S. aureus* can fuel malignant T-cell proliferation in vivo^40^. The present study was undertaken to determine whether and how clinical isolates, and SE modulate FOXP3 expression in malignant cells from SS patients.

## Materials and methods

### Antibodies and reagents

IL-2- and IL-15-blocking antibodies were purchased from R&D Systems (Minneapolis, MN). Erk1/2 antibodies were obtained from Santa Cruz Biotechnology (Santa Cruz, CA). FOXP3 (236 A/E7) for western blotting was from eBioscience (San Diego, CA, USA). Fluorochrome-conjugated CD3, CD4, CD7, CD8, CD19, CD25, CD26, pY-STAT5, FOXP3, and respective fluorochrome-conjugated isotype control Abs used for FACS were provided by Biolegend (San Diego, CA, USA) and BD Biosciences (San Jose, CA, USA). The SE (staphylococcal enterotoxin A (SEA), SEB, SEC2, SED, and SEI) from Toxin Technology (Sarasota, FL, USA), Propidium iodide was from Thermo Fisher Scientific (Waltham, MA, USA), and Fixable Viability Stain Dye eFluor780 from eBioscience. SEA mutants were generously provided by Active Biotech (Lund, Sweden).

### Patients and isolation of *S. aureus* bacteria

Malignant and nonmalignant cells were isolated from the blood of patients diagnosed with SS in accordance with the World Health Organization/European Organization for Research and Treatment of Cancer classification^41^. See Supplementary Table 1 for patient characteristics. Malignant cells typically lack the expression of cell surface marker CD26 and/or CD7 (ref. ^2^). Accordingly, T cells were identified as malignant (CD4+, CD7dim/−, and CD26dim/−) and nonmalignant (CD4+/CD7+, and CD26+). Bacterial isolates were collected from CTCL patients using swabs wetted with 0.1% Triton X-100 in 0.075 M phosphate buffer, transferred to Stuart transport medium, and cultivated on blood agar overnight at 37 °C at 5% carbon dioxide.

In accordance with the Declaration of Helsinki, all samples were obtained with informed consent after approval by the Committee on Health Research Ethics (H-16025331).

### Cell lines

The malignant T-cell line SeAx and the nonmalignant T-cell line, MF1850, were established from patients diagnosed with CTCL (ref. ^42^), and cultured in media supplemented with 10% human serum (HS medium) and IL-2. Cell lines were tested for mycoplasma contamination. Prior to experimental setup, the CTCL cell lines were starved overnight in HS medium without IL-2.

### Detection of SE in bacterial isolate supernatants

The presence of SE in bacterial cultures was examined using the RIDASCREEN SET A, B, C, D, E kit (R-Biopharm, Darmstadt, Germany), with a toxin detection limit of 0.25 ng/mL and in accordance with the manufacturer’s instructions.

### RNA purification, complementary DNA synthesis, and qPCR

Total cellular RNA was purified and reverse transcribed into complementary DNA as previously described^43^. Quantitative polymerase chain reaction (qPCR) was performed using the TaqMan assay from Thermo Fisher Scientific in accordance with the manufacturer’s instructions, and the samples were analyzed on a LightCycler480 II instrument (Roche).

### Cell isolation, flow cytometry, and cell sorting

Peripheral blood mononuclear cells (PBMCs) were isolated from the blood of SS patients by lymphoprep density-gradient centrifugation (Stemcell technologies) and^1^ used directly for flow cytometric analysis^2^, cultured in HS medium with phosphate-buffered saline (PBS) or SE, or^3^ sorted by FACS using FACSAria-II (BD Biosciences) into populations of malignant and nonmalignant cells based on CD4 and CD26 surface expression, and then mono- or co-cultured in HS medium with PBS or SE. Purity of the sorted malignant and nonmalignant cells was 99% and 95%, respectively. In experiments in which co-cultured SeAx and MF1850 cells were sorted, the SeAx cells were stained prior to culture with 1 uM carboxyfluorescein succinimidyl ester (CFSE). The CFSE-positive (SeAx) and CFSE-negative (MF1850) cells were sorted by FACSAria resulting in a purity of 98%. Data acquisition and flow cytometric analysis were performed on LSR Fortessa flow cytometers (BD Biosciences) using FlowJo software (Tree Star, Ashland, OR).

### Western blotting

Cells were rapidly pelleted and lysed in ice-cold lysis buffer (1% NP-40, 20 mM TRIS-HCl pH 8.0, 140 mM NaCl, 10% glycerol, and the following inhibitors: 1 mM phenylmethylsulfonyl fluoride, 1 mM Na3VO4, 10 mM NaF, 1 mM iodioacetamide, 5 mM ethylenediaminetetraacetic acid, and 7.5 μg/ml aprotinin) for 30 min on ice. Total cell lysates were boiled in reducing sodium dodecyl sulfate (SDS) sample buffer and subjected to SDS–polyacrylamide gel electrophoresis followed by electrophoretic transfer to a nitrocellulose (NC) membrane. Afterward, the NC membrane was blocked with 3% milk in PBS-T for 1 h and then incubated with primary Abs overnight. Subsequently, the NC membrane was incubated with HRP-conjugated secondary Abs for 1 h (DAKO). Blots were evaluated using enhanced chemiluminescence according to the manufacturer’s instructions. To ensure equal loading, the total protein concentration of each sample was determined by Bio-Rad Protein Assay (Bio-Rad; Hercules, CA, USA).

### Transient transfections

A total of 2 × 10^6^ cells per sample were transfected with siRNA against STAT5 or nontargeting control (ON-TARGETplus SMARTpool; Thermo Scientific, Lafayette, CO, USA). Pellets were resuspended in 100 mL of Transfection solution (Ingenio Electroporation Solution; Mirus Bio, Madison, WI, USA) in the presence of 0.25 mM of the respective siRNAs, and transfected with an Amaxa Nucleofector (Lonza, Cologne, Germany).

### Statistics

For statistical analysis, a two-tailed Student’s *t*-test with a significance level of *P* = 0.05 was applied. Error bars represent standard error of the mean.

## Results

### SE induce FOXP3 and CD25 upregulation in malignant cells

**Fig. 1:**
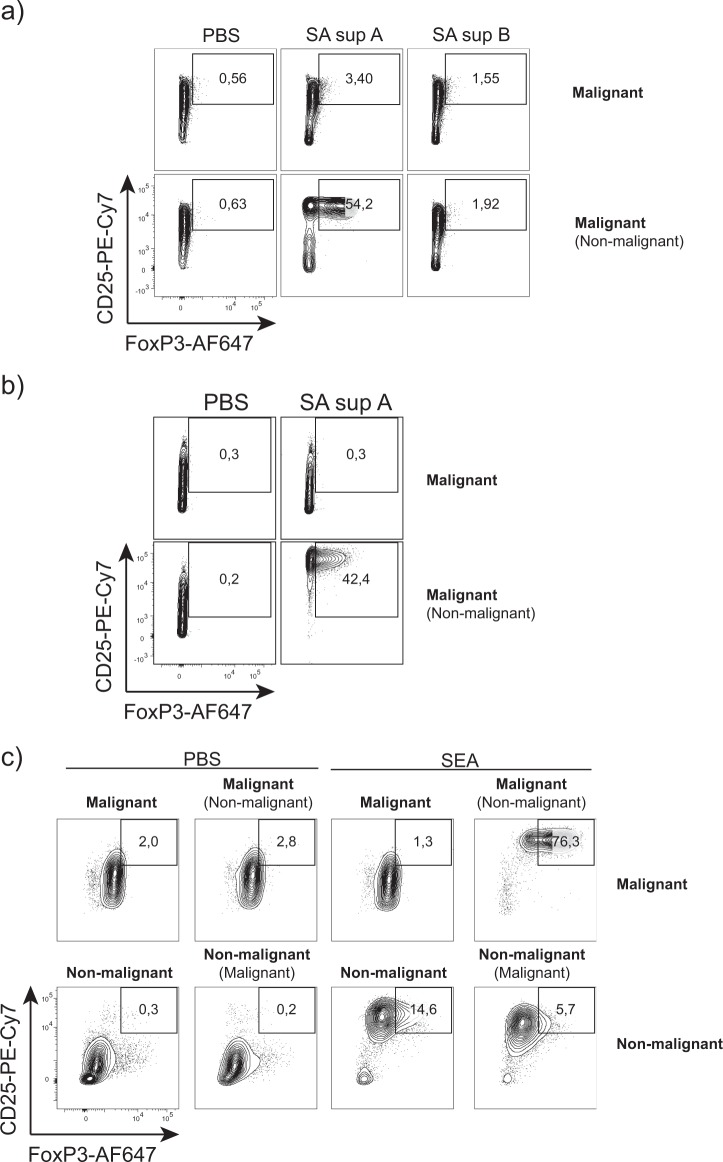
SE induce FOXP3 and CD25 upregulation in malignant cells. **a** Malignant cells (SeAx) were cultured alone or in the presence of nonmalignant cells (MF1850) with vehicle (PBS), staphylococcal enterotoxins (SE), or with either a SE-positive (SA sup A) or SE-negative (SA sup B) supernatant from clinical *S. aureus* isolates for 24h. Contour plots show gated malignant cells. **b** Malignant cells (SeAx) were cultured alone or in the presence of nonmalignant cells (MF1850) with vehicle (PBS) or with a SE-positive supernatant from a clinical *S. aureus* isolate (SA sup A) for 72h. Contour plots show gated malignant cells. **c** Malignant cells (SeAx) and nonmalignant cells (MF1850) were cultured alone or in the presence of nonmalignant cells and malignant cells, respectively, with either vehicle (PBS) or SEA for 48h. Contour plots show gated malignant cells in upper row and nonmalignant cells in bottom row.

Prior to flow cytometric analysis for FOXP3 and CD25 expression, the malignant SS T-cell line SeAx, was cultured with and without a nonmalignant T-cell line (MF1850) in the presence or absence of supernatants from two clinical *S. aureus* isolates that were either positive or negative for SE. Figure 1a shows FOXP3 and CD25 expression in malignant cells, following 24 h of monoculture (Fig. 1a, upper row) and co-culture with nonmalignant cells (Fig. 1a, lower row) with SE-positive (SA sup A) and -negative (SA sup B) supernatants, and PBS as control (Fig. 1a). Strikingly, the SE-positive supernatant induced a profound upregulation of FOXP3 and CD25 expression by malignant cells co-cultured with nonmalignant cells (Fig. 1a, lower, middle). In contrast, the SE-positive supernatant had little effect on malignant cells grown in monoculture (Fig. 1a, upper, middle), in agreement with our published observations^20,38^. A similar expression pattern was obtained after 3 days of culture with *S. aureus* supernatants (Fig. 1b). Applying SEA yielded essentially identical results (Fig. 1c). Importantly, SEA induced a very high co-expression of FOXP3 and CD25 in malignant cells co-cultured with nonmalignant cells (Fig. 1c, upper right), as compared to co-cultures without SEA, and monocultures with and without SEA (Fig. 1c, upper left). In nonmalignant cells, SEA induced strong CD25 expression, but only weak FOXP3 expression when co-cultured with malignant cells (Fig. 1c, lower right), whereas nonmalignant cells in monoculture responded to SEA with a moderate upregulation of FOXP3, when compared to cultures without SEA (Fig. 1c, lower middle and left). FOXP3/CD25 co-expression was considerably higher in malignant cells, when compared to nonmalignant cells in SEA-stimulated co-cultures (Fig. 1c, upper versus lower right). Taken together, malignant cells can be induced by SE to express high levels of both CD25 and FOXP3, if nonmalignant cells are present. These findings support a model where SE induces crosstalk between malignant and nonmalignant cells, which in turn affects the phenotype of the malignant cells. These findings prompted us to investigate whether SE could also stimulate FOXP3 expression in primary malignant cells from SS patients, following co-culture with nonmalignant cells.

### Primary SS cells upregulate FOXP3 after stimulation with SE

**Fig. 2:**
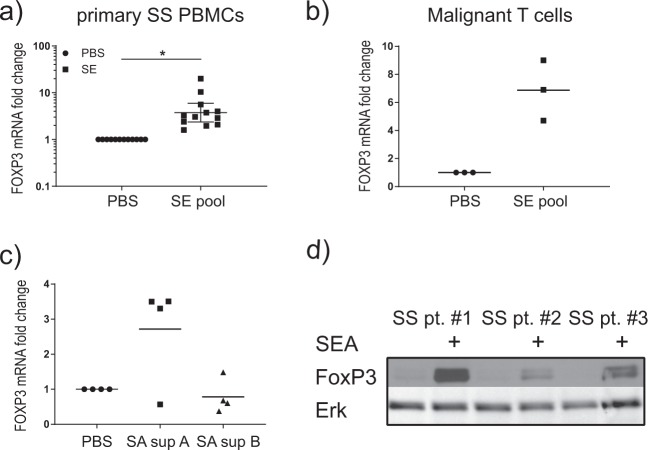
Primary SS cells upregulate FOXP3FOXP3 after stimulation with SE. **a** PBMCs from Sézary syndrome (SS) patients were cultured in the presence of vehicle (PBS) or a pool of *S. aureus* enterotoxins (SE) (SEA, SEB, SEC2, SED, and SEI) for 24h. *FOXP3* expression was determined by qPCR. In each sample, the level of *FOXP3* mRNA was normalized to that of *GAPDH* mRNA, and it is depicted as fold change compared to samples with PBS with geometric mean and 95% confidence interval. Student’s *t*-test was applied on delta CT values, *p*<0.05 (*n*=12). **b** PBMCs from SS patients were cultured in the presence of vehicle (PBS) or a pool of SE (SEA, SEB, SEC2, SED, and SEI) for 24h, and then sorted by FACS into malignant and nonmalignant cells. *FOXP3* expression was determined by qPCR. In each sample, the level of *FOXP3* mRNA was normalized to that of *GAPDH* mRNA and it is depicted as fold change compared to samples treated with PBS only. **c** PBMCs from SS patients were cultured in the presence of vehicle (PBS), or with either a SE-positive supernatant (SA sup A) or a SE-negative supernatant (SA sup B) from clinical *S. aureus* isolates for 72h. *FOXP3* expression was determined by qPCR. In each sample, the level of *FOXP3* mRNA was normalized to that of *GAPDH* mRNA and it is depicted as fold change compared to samples with PBS (*n*=4). **d** PBMCs from three SS patients were cultured in the presence of vehicle (PBS) or SE for 24h. *FOXP3* expression was determined by western blotting.

**Fig. 3:**
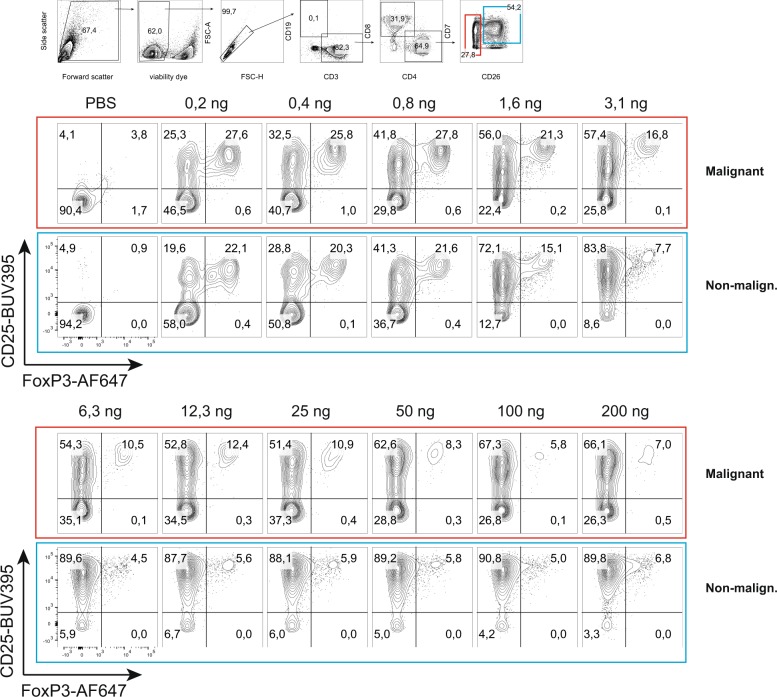
Maximal FOXP3 induction at suboptimal concentrations of SE. FOXP3 and CD25 expression is shown for gated SS cells (red border), and nonmalignant cells (blue border).

Initially, we measured *FOXP3* mRNA in PBMCs from SS patients cultured with a pool of SEs, including SEA, SEB, SEC2, SED, and SEI. As shown in Fig. 2a, a pool of SEs induced a significant increase in *FOXP3* mRNA expression in primary PBMCs from SS patients. Next, we measured *FOXP3* mRNA in sorted malignant cells after culture of PBMCs from SS patients stimulated with a pool of SEs. Figure 2b shows that primary malignant cells did indeed upregulate *FOXP3* following co-culture with SEs and nonmalignant cells. Supernatants from SE containing clinical isolates also induced *FOXP3* expression in primary PBMCs from three out of four SS patients (Fig. 2c). Likewise, FOXP3 protein expression was detected in PBMCs from three SS patients stimulated with SEA (Fig. 2d). In accordance, SEA induced FOXP3 expression in CD25-positive malignant and nonmalignant T cells, as judged from flow cytometry analysis of primary SS cells (Fig. 3, upper PBS versus 0.2–0.8 ng/ml). Higher concentrations of SEA did not further increase the fraction of FOXP3-positive cells. On the contrary, the fraction of FOXP3-positive T cells gradually declined in response to SEA concentrations above 0.8 ng/ml, suggesting that an increased concentration did not increase a stronger FOXP3 response in primary SS cells (Fig. 3, lover part).

### FOXP3 upregulation in malignant cells is dependent on IL-2/STAT5 signaling

**Fig. 4:**
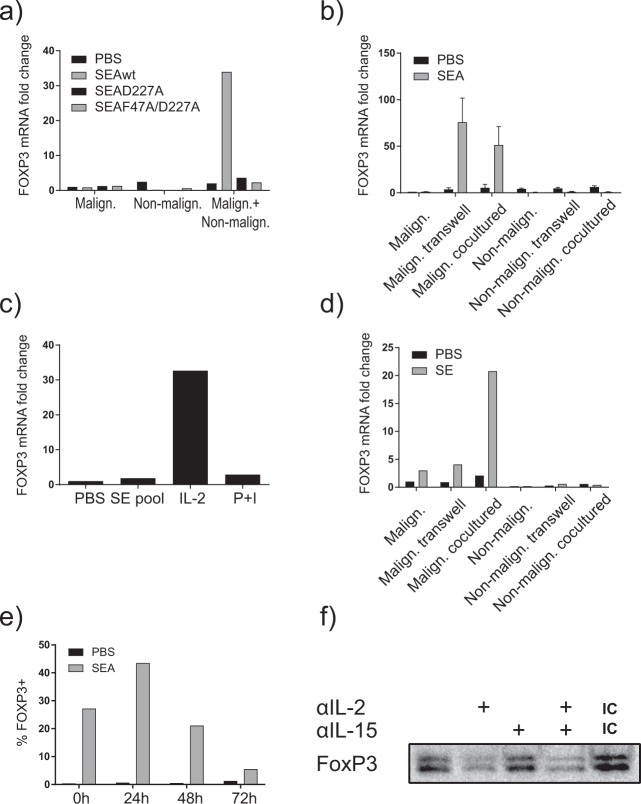
FOXP3 upregulation depends on MHC class II and IL-2. **a** Malignant cells (SeAx) and nonmalignant (MF1850) T cells were cultured alone or together and in the presence of either vehicle (PBS), *S. aureus* enterotoxin A (SEA) wild type (SEAwt), or MHC-II binding-deficient SEA mutants (SEAD227A or SEAF47A/D227A). *FOXP3* expression was determined by qPCR. In each sample, the level of *FOXP3* mRNA was normalized to that of *GAPDH* mRNA and it is depicted as fold change compared to malignant cells with PBS. **b** Malignant cells (SeAx) and nonmalignant (MF1850) T-cell lines were cultured alone, co-cultured separated by transwells, and cultured in the presence of vehicle (PBS) or SEA for 24h. The co-cultured malignant and nonmalignant cells were sorted by FACS, and the relative level of *FOXP3* and *GAPDH* mRNA were determined in all samples by qPCR. In each sample, the level of *FOXP3* mRNA was normalized to that of *GAPDH* mRNA and is depicted as fold change compared to mono-cultured malignant cells with PBS. “Malign. transwell” signifies *FOXP3* expression in malignant cells in transwell with nonmalignant cells and vice versa for “nonmalign. transwell”. “Malign. co-cultured” signifies *FOXP3* expression in malignant cells co-cultured with nonmalignant cells and vice versa for “nonmalign. co-cultured”; (*n*=3). **c** FACS sorted primary Sézary syndrome (SS) cells were cultured in the presence of vehicle (PBS), SE, IL-2, or PMA+Ionomycine for 24h. *FOXP3* expression was determined by qPCR. In each sample, the level of *FOXP3* mRNA was normalized to that of *GAPDH* mRNA and it is depicted as fold change compared to malignant cells with PBS. **d** Primary SS cells and nonmalignant (MF1850) T-cell lines were either cultured alone, co-cultured separated by transwells, or cultured together with vehicle (PBS) or a pool of SE (SEA, SEB, SEC2, SED, and SEI) for 24h. The co-cultured malignant cells and nonmalignant cells were sorted by FACS, and the relative level of *FOXP3* and *GAPDH* mRNA were determined in all samples by qPCR. In each sample, the level of *FOXP3* mRNA was normalized to that of *GAPDH* mRNA and it is depicted as fold change compared to mono-cultured malignant cells with PBS. “Malign. transwell” signifies *FOXP3* expression in malignant cells in transwell with nonmalignant cells and vice versa for “nonmalign. transwell”. “Malign. co-cultured” signifies *FOXP3* expression in malignant cells co-cultured with nonmalignant cells and vice versa for “nonmalign. co-cultured”. **e** Malignant cells (SeAx) and nonmalignant (MF1850) T cells were co-cultured for 3 days in the presence of vehicle (PBS) or SEA, and then spun down and washed three times and resuspended in fresh media. FOXP3 expression in malignant cells was analyzed by flow cytometry after 0, 24, 48, and 72h. **f** Malignant cells (SeAx) and nonmalignant (MF1850) T cells were cultured together, and in the presence of SEA and either anti-human IL-2, anti-human IL-15, a combination thereof, or isotype control. FOXP3 expression was determined by western blotting.

Having established that malignant cells can upregulate *FOXP3* expression in response to SE if nonmalignant cells are present, we decided to study in detail the crosstalk and signaling pathways involved. As shown in Fig. 4a, mutations in the MHC class II binding site of SEA (D227A and F27A) effectively abrogated the SEA-induced *FOXP3* expression, indicating that *FOXP3* expression was dependent on the binding of SEA to MHC class II molecules. To examine whether the SE-induced *FOXP3* expression in malignant cells in co-culture involved soluble factors and/or cell–cell contact with nonmalignant cells, we applied monocultures and co-cultures as above with and without inserted semipermeable membranes. These membranes allow exchange of soluble factors, such as cytokines, but bar cell passage. As shown in Fig. 4b, SEA induced comparable *FOXP3* expression in malignant cells in co-culture with and without semipermeable membranes separating them from nonmalignant cells, indicating that soluble factors are sufficient in SEA-induced *FOXP3* expression in malignant cells, whereas cell–cell contact between malignant and nonmalignant cells was not required. As we have previously reported that SEA induced IL-2 expression by nonmalignant CD4^+^ T-cell lines^20^, we examined whether exogenous IL-2 or mitogenic stimuli (PMA in combination with Ionomycin) could induce *FOXP3* in primary malignant cells. As shown in Fig. 4c, IL-2 induced *FOXP3* expression in primary malignant cells, whereas PMA and Ionomycin did not. In accordance with the findings above, SE had no effect on *FOXP3* expression by malignant cells per se, i.e., in the absence of nonmalignant cells. However, following co-culture with SE and allogeneic nonmalignant cells, primary malignant cells displayed SE-induced *FOXP3* expression (Fig. 4d). Thus, the addition of nonmalignant cells together with SE caused strong *FOXP3* upregulation in otherwise nonresponsive primary malignant cells. Taken together these findings suggest that SE triggered nonmalignant cells to release soluble factors, which in turn induced *FOXP3* expression in malignant cells. As SEA induced a transient expression of IL-2 by nonmalignant T cells in our co-culture system^20^, we addressed whether induction of FOXP3 was also transient and mediated by IL-2. Accordingly, co-cultures were pretreated with and without SEA for 72 h prior to extensive washing, and subsequent co-culture for additionally up to 72 h. As shown in Fig. 4e, pretreatment with SEA induced a substantial fraction of FOXP3-positive cells (28%) compared with <1% in untreated (PBS) controls (Fig. 4a; 0 h, left lane). Twenty-four hours after pretreatment with SEA, the fraction of FOXP3-positive cells increased further to above 40% (Fig. 4e, 24 h) followed by a gradual decline to <10% FOXP3-positive malignant T cells at 72 h (Fig. 4e, 72 h). An IL-2 neutralizing antibody inhibited SE-induced FOXP3 expression in malignant cells following co-culture with nonmalignant cells (Fig. 4f), whereas an IL-15 neutralizing antibody had little effect as judged by western blotting, which detects multiple isoforms of FOXP3 (ref. ^19^).

**Fig. 5:**
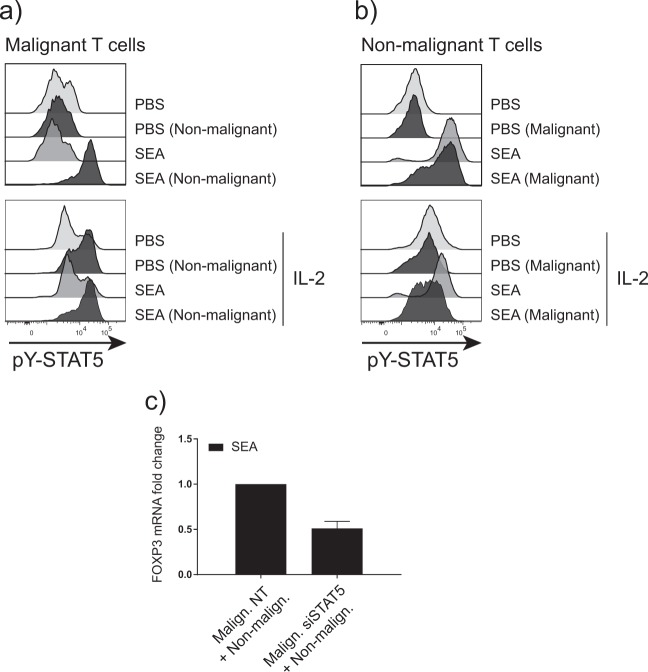
FOXP3 upregulation depends on IL-2/STAT5 signaling. **a** Malignant cells (SeAx) were cultured alone or together with nonmalignant cells (MF1850), and in the presence of vehicle (PBS) or *S. aureus* enterotoxin A (SEA) with and without exogenous IL-2 for 48h. pY-STAT5 was determined by flow cytometry. **b** Nonmalignant cells (MF1850) were cultured alone or together with malignant cells (SeAx), and in the presence of vehicle (PBS) or SEA with and without exogenous IL-2 for 48h. pY-STAT5 was determined by flow cytometry. **c** Malignant cells (SeAx) were treated with nontargeting siRNA control or siRNA targeting STAT5, and cultured with nonmalignant (MF1850) in the presence of SEA. *FOXP3* expression was determined by qPCR. *FOXP3* mRNA was normalized to that of *GAPDH* mRNA and it is depicted as fold change compared to nontargeting siRNA control.

Since FOXP3 is a well-known target of STAT5 in malignant cells^44^ and STAT5 is downstream of IL-2 signaling^45^, we investigated STAT5 activation in malignant and nonmalignant cells cultured with and without SEA. Figure 5a shows that SEA induced strong STAT5 activation in malignant cells following co-culture with nonmalignant cells measured by intracellular flow cytometry, whereas SEA had no effect on STAT5 activation in monocultures of malignant cells (Fig. 5a, upper). In contrast, SEA induced STAT5 activation in monocultures of nonmalignant cells, which was largely unaffected by the presence of malignant cells (Fig. 5b, upper). Exogenous IL-2 induced a modest STAT5 activation in malignant cells, which was further upregulated in co-cultures (Fig. 5a, lower). IL-2 also induced Stat5 activation in nonmalignant cells—irrespective whether malignant cells were present in the culture (Fig. 5b, lower). siRNA-mediated knockdown of STAT5 in malignant cells decreased *FOXP3* expression by roughly 50% (Fig. 5c), confirming previous data that *FOXP3* transcription is at least partly mediated by STAT5 in malignant cells^19^.

## Discussion

Here, we show that SE-positive supernatants from *S. aureus* CTCL skin isolates, as well as SE can induce expression of FOXP3 in both primary malignant cells and in an immortalized patient-derived T-cell line, when cultured in the presence of nonmalignant cells. Since malignant cells alone were largely unresponsive to SE, our findings indicate that induction of FOXP3 expression in malignant cells is indirect and mediated by signals derived from nonmalignant cells. In support, transwell experiments showed that soluble factors were involved, whereas direct cell–cell contact between malignant and nonmalignant cells was not required for SE-induced FOXP3 expression in malignant cells. IL-2 is a well-established inducer of FOXP3 in T cells^46^ and importantly, blockage of IL-2 by neutralizing antibodies strongly inhibited FOXP3 induction by SE in co-cultures. STAT5 is an IL-2-activated transcription factor involved in induction of FOXP3 expression^44^. In accordance, siRNA-mediated knockdown of STAT5 inhibited SE-induced FOXP3 expression by malignant cells in co-cultures. Collectively these findings support a model^20,38^, where SE induces nonmalignant cells to produce and release IL-2, as well as other soluble factors that in turn upregulate FOXP3 expression in malignant cells. This model implies that nonmalignant cells can respond to SE, which was indeed the case as seen in monocultures of nonmalignant cells.

It may come as a surprise, that nonmalignant cells respond directly to SE, since it is well known that SE stimulation of murine T cells (expressing an appropriate T cell receptor (TCR) V*β* chain) depends on the presence of MHC class II-positive antigen-presenting cells (APCs). In contrast to murine T cells, however, human T cells express MHC class II molecules upon activation^47^. Moreover, nonmalignant cells lines from CTCL patients and CD4 T-cell lines from healthy individuals constitutively express MHC class II molecules^48^ and are able to present SE to other T cells^48^. MHC class II expressing T cells respond to very low concentrations of SE. In accordance, mutations in the MHC class II binding site of SEA abrogated the ability to induce FOXP3 expression in malignant cells in co-cultures. As MHC class II molecules also function as signaling molecules capable of inducing tyrosine kinase activity, activation of PLC-γ, and expression of IL-2 receptors in co-activated human T cells^49^, it is possible that MHC class II and TCR act synergistically in SE-mediated activation of nonmalignant cells. In contrast to cultures of malignant and nonmalignant cell lines, primary PBMC cultures from healthy individuals contain MHC class II-positive APCs, such as dendritic cells, monocytes, and B cells, all of which are able to present SE to T cells. Although the numbers of these cell types are often reduced to a varying degree in SS patients, it is likely that APCs in PBMC cultures from SS patients contribute to SE presentation, because even small numbers of APCs are highly potent to present SE to T cells^50^. We suspect that this may also account for some of the variation in SE-mediated activation of malignant cells, as the number of cells that are able to present SE must vary between SS patients with different tumor loads.

Since the original findings by Berger and colleagues that CTCL involved malignant proliferation of Treg cells, several studies have investigated whether or not malignant cells displayed a FOXP3^+^ (refs. ^19,24–29^) phenotype in peripheral blood of SS patients. Using different methods and cohorts of patients, investigators have come to very different conclusions. For instance Klemke et al. only detected FOXP3^+^ T cells in a minority of patients, but with a bimodal expression pattern, i.e., some patients displayed high numbers of FOXP3^+^ T cells, while others had reduced numbers compared to controls^25^. In contrast, Capriotti et al. reported that PBMCs from 30% of SS patients expressed FOXP3 and interestingly, that those patients with FOXP3 expression had a significantly worse prognosis than patients without^26^. In accordance, our group has found that the malignant cells from 8 out of 15 SS patients stained positive for FOXP3 (ref. ^19^). Heid et al. obtained comparable results^27^, whereas several others reported on reduced or completely lacking expression of FOXP3 in SS patients compared to healthy donors^24,28,29^. Kasprzycka et al.^17^ obtained evidence of a heterogeneous FOXP3 expression in malignant cells in lesional skin and proposed a hypothesis, where cytokines in the tumor microenvironment drive the heterogeneous FOXP3 expression in malignant cells^17^. Since the majority of SS patients with advanced disease have a colonization or infection of their skin with SE-producing *S. aureus*^51^, and SE induce FOXP3 expression in malignant cells as shown here in vitro, it is likely that SE may also induce FOXP3 expression in vivo through induction of cytokine expression by nonmalignant cells in the tumor microenvironment.

Accordingly, differences in FOXP3 expression by malignant cells between SS patients and cohorts of patients may reflect differences in skin colonization and infection with SE-producing *S. aureus*. This mechanism also provides a possible explanation for some of the disease heterogeneity between SS patients and within individual SS patients^11,30,52,53^, as well the reported discrepancies between different cohorts of patients. If confirmed in independent cohorts, enhanced FOXP3 expression by malignant T cells may have potential as a marker of infection-induced immune deregulation. Moreover, we believe that our findings lend support to a clinical rationale for aggressively treating *S. aureus* infections in selected SS patients, in order to dampen inflammation and limit any potential immune-regulatory effects exerted by activated malignant T cells.

In conclusion, we show for the first time ever that *S. aureus* SE can induce FOXP3 expression in malignant cells obtained from SS patients. Therefore, we propose that SE-producing *S. aureus* in the tumor microenvironment modulate the phenotype of malignant cells in vivo.

## Supplementary information

Supplementary table S1

aj-checklist
