# Supplementary material for: Staphylococcus aureus enterotoxins induce FOXP3 in neoplastic T cells in Sézary syndrome
Source: Blood Cancer J. 2020 May 14;10(5):57. doi: 10.1038/s41408-020-0324-3 (PMC7225173; doi:10.1038/s41408-020-0324-3)
Supplement: Supplementary file 1 — Supplementary table S1 [file 41408_2020_324_MOESM1_ESM.pdf]

Supplementary figure S1

Patient	Sex	Age, y	Diagnosis	ISCL classification	Percent CD4+ lymphocytes	CD4:CD8 ratio	ECP treatment
pt #1	M	na	SS	B2	62%	2,0	ECP
pt #2	M	na	SS	B2	89%	12,5	ECP
pt #3	M	65	SS	B2	82%	14,2	ECP
pt #4	M	69	SS	B2	87%	14,0	ECP
pt #5	F	na	SS	B2	na	na	ECP
pt #6	M	67	SS	B2	70%	16,2	ECP
pt #7	F	na	SS	B2	93%	95,2	ECP
pt #8	M	79	SS	B2	90%	56,6	ECP
pt #9	M	76	SS	B2	93%	411,5	ECP
pt #10	M	85	SS	B2	31%	0,5	ECP
pt #11	M	74	SS	B2	72%	4,6	ECP
pt #12	F	58	SS	B2	85%	27,3	ECP

Patient characteristics. ECP, extracorporeal photopheresis; SS, Sézary syndrome; na, not available
